# Knockdown of LncRNA SNHG1 Suppresses Corneal Angiogenesis by the Regulation of miR-195-5p/VEGF-A

**DOI:** 10.1155/2021/6646512

**Published:** 2021-10-19

**Authors:** Xiaofeng Hu, Hongru Xing, Xiaofeng Wang, Lei Du, Yihong Huang, Youguo Hao, Zhibin Shen

**Affiliations:** ^1^Department of Ophthalmology, Beijing Chaoyang Hospital, Capital Medical University, Beijing 100020, China; ^2^Neuroelectrophysiology Department, People's Hospital of Shanxi Province, No. 29 Shuangtasi Street, Taiyuan 030000, Shanxi, China; ^3^Department of Neurosurgery, Weinan Central Hospital, Shengli Road, Weinan 714000, Shanxi Province, China; ^4^Department of Neurosurgery, General Hospital of Ningxia Medical University, No. 804 Shengli Street, Xingqing District, Yinchuan 750000, Ningxia Hui Autonomous Region, China; ^5^Department of Neurology, The Affiliated Houjie Hospital, Guangdong Medical University, No. 21 Hetian Avenue, Dongguan 523000, Guangdong, China; ^6^Department of Rehabilitation, Shanghai Putuo People's Hospital, No. 1291 Jiangning Road, Shanghai 200060, China; ^7^Department of Ophthalmology, Dazhou Central Hospital, Dazhou, Sichuan 635000, China

## Abstract

LncRNA SNHG1 (SNHG1) has been widely studied as the causative factor of angiogenesis and proliferative agent in gastric, lung, cervical, and hepatocellular carcinomas. However, its significance of angiogenesis and progression of corneal neovascularization (CRNV) is least understood. This study focuses on the molecular mechanisms followed by SNHG1 to establish CRNV and its angiogenesis. Bioinformatics analysis to identify potential miRNA targets of SNHG1 and vascular endothelial growth factor A (VEGF-A) was conducted using StarBase and was subsequently confirmed by the luciferase reporter assay. Relative quantitative expression of SNHG1 in human umbilical vein endothelial cells (HUVECs) was detected through qRT-PCR and western blot analysis. Cell proliferation was detected through CCK-8 assay, whereas migratory abilities of the cells were determined with transwell assay. A capillary-like tube formation assay was performed to detect the tube formation ability of the cells. Following this, relative expression of miR-195-5p and VEGF-A was determined through qRT-PCR and western blot analysis. Results from the experiments manifested upregulated levels of SNHG1 and VEGF-A in HUVECs and CRNV tissues as compared with the control group, whereas downregulated levels of miR-195-5p were measured in the CRNV tissues and HUVECs, suggesting the negative correlation between lncRNA and miRNA. Overexpressed vascular endothelial growth factor promoted cell proliferation and tube formation; however, its silencing leads to inhibition in angiogenesis and proliferation. Potential binding sites of SNHG1 showed miR-195-5p as its direct target and SNHG1 as a sponge for this miRNA. Knockdown and downregulated levels of SNHG1 showed a notable decrease and inhibition in angiogenesis and migration of CRNV cells. The study showed that SNHG1 inhibition significantly reduced cell proliferation, migration, and tube formation in HUVECs transfect with lncRNA SNHG1. Mechanistic insights into the SNHG1 showed that SNHG1 acts as a sponge for miR-195-5p and upregulates the levels of VEGF-A.

## 1. Introduction

The cornea is avascular and transparent and can easily be identified with its unique framework of capillaries around the corneal limbus. The pathological condition that disturbs the refraction abilities and transparency by the development of blood vessels inside the cornea is named corneal neovascularization (CRNV) [1]. CRNV often occurs because of hypoxia, sudden traumas, inflammation and toxicity, or nutritional deficiencies and is largely affected by the angiogenesis factors [2, 3]. Recent studies have demonstrated a lot of research in the diagnosis, molecular mechanisms, and disease progression of CRNV; however, many factors still need to be investigated for their role in CRNV angiogenesis.

Among other determinants of CRNV development, lncRNAs have been widely studied in recent years for their roles in disease. LncRNAs are stretches of RNAs that are characterized as post-transcriptional regulators of gene expression. Upregulated levels of lncRNAs have been widely explored in oxidative stress, angiogenesis, and metastasis [4]. For example, lncRNAs as oncogenes in glioblastomas have been evaluated through xenografts [5]. Similarly, in hepatocellular carcinomas, aberrant expressions and upregulated levels of several lncRNAs were elucidated [6]. However, very few studies report the role and significance of lncRNAs in CRNV progression and angiogenesis. Particularly, the role and underlying mechanism of LncRNA SNHG1 (SNHG1) have not been largely described.

Similarly, microRNAs represent another class of RNAs that can directly control protein synthesis via post-transcriptional regulation. LncRNAs disrupt the balance of gene expression by sponging several miRNAs and thus lead to angiogenesis [7]. Following research, SNHG1 was hypothesized to directly target miR-195-5p and increase VEGF-A levels. Thus, lncRNAs act as a sponge for microRNAs and disrupt their balance to maintain the biological functions of gene expression.

Vascular endothelial growth factor (VEGF) is widely reported to increase vascular permeability and enhances endothelial cell proliferation through its mitogen-based activities [8]. Accumulating research studies have shown abnormal expression of VEGF in endothelial cells and epithelium of the cornea in the CRNV patients as compared to normal corneal cells. Its critical role has been demonstrated in CRNV angiogenesis through the binding of its receptors Flk-1 and Flt-1 causing the increase of blood vessels in permeability. The following study was aimed to study the effects of VEGF and SNHG1 overexpression in the HUVECs and the significance of miR-195-5p in controlling the CRNV angiogenesis.

## 2. Materials and Methods

### 2.1. Ethics Statement

Ethical consent was obtained from the local Lab Animal Handling Committee of the Central Hospital of Dazhou, and all the experiments were conducted keeping in focus the World Medical Association Declaration described for animals in Ophthalmic Research (approval number: KE-Y-2020-03-18). Animals were kept in proper 12 h dark–light cycles, and surgical samples were collected following the Declaration of Helsinki. Informed consent was also obtained from all subjects to be included in the research.

### 2.2. Human Tissue Samples

In this study, 20 patients within the screening range were being procured from CNV patients with corneal transplantation, and 12 healthy corneal rims were obtained from the Ophthalmology Section of the Central Hospital of Dazhou. According to the criteria of corneal angiogenesis, angiogenesis existed in the periphery of the cornea more than 2 mm, which is considered to be corneal angiogenesis. Among all patients, 12 cases complied with the requirements and underwent keratectomy. Samples were snap-frozen at −80°C for further analysis.

### 2.3. Cell Culture

Endothelial basal medium containing hydrocortisone, epidermal growth factor, antibiotic gentamycin, bovine brain extract, and 10% FBS (R&D Systems, USA) was used to culture HUVECs (PromoCell, Germany) at 37°C, 95% humidity, and 5% CO_2_. Human VSMCs were procured from ATTC and maintained in Hams F12 medium (Kaighn's modified) complemented with FBS (10%) and VSMC growth medium (50%).

### 2.4. Quantitative Real-Time (qRT-PCR)

Total tissue RNA was retrieved with TRIzol reagent (A33250, Invitrogen) focusing the manufacturer's guidelines. RNA was quantified with a spectrophotometer (Thermo Scientific). First-strand cDNA Synthesis kit (04379012001; Roche Life Sciences, Mannheim, Germany) was used to synthesize RNA into cDNA according to the standard kit protocol. A qScript One-Step RT-qPCR kit (95057-050; Quantabio, Beverly, MA, USA) was utilized to perform qRT-PCR in the PCR system (LineGene, Latvia). Primer set for VEGFA comprising F-5′-GAATGGGGAGCCCAGAGT-3′and R-5′-CCACTTCGTGATGATTCTGC-3′, miR-195-5p comprising F-5′-GGCGTCGTATCCAGTGCAAT-3′ and R-5′-GTCGTATCCAGTGCGTGTCG-3′, SNHG1 primers F-5′-AGGCTGAAGTTACAGGTC-3′ and R-5′-TTGGCTCCCAGTGTCTTA-3′, U6 comprising F-5′-CTCGCTTCGGCAGCACA-3′ and 5′-AACGCTTCACGAATTTGCGT-3′, and GAPDH comprising F-5′CCCACTCCTCCACCTTTGAC-3′ and R-5′CATACCAGGAAATGAGCTTGACAA-3′ were used according to reference publication. GAPDH and U6 served as internal controls following quantification of relative gene expression through the 2^−ΔΔCT^ calculation method [9].

### 2.5. Cell Transfection Assay

HUVECs cells transfection was done with lncRNA SNHG1 and miR-195-5p as per manufacturer's instructions. Transfection was done by culturing HUVECs in a 96-well plate at the concentration of 1 × 10^6^ cells/well. Small interfering RNAs such as siSNHG1, siVEGF-A, and siNC were available from Invitrogen. Moreover, controls, inhibitors, and mimics for miR-195-5p were procured from Gene Pharma (China). Overexpressing VEGF-A plasmids were construed with pcDNA3.1 plasmid (Thermo Fisher Scientific, USA). Lipofectamine (LFN) 3000 reagent (Invitrogen, USA) was used only for the transfection assay following the guidelines by manufacturer's protocol, and efficiency of transfection was measured by qRT-PCR [10].

### 2.6. Cell Counting Kit Bioassay (CCK-8)

The cell viability was tested with the CCK-8 kit (Dojindo) following the manufacturer's guidelines. Cells were seeded into a 96-well plate (3 × 10^4^ cells/well) and incubated for 24, 48, 72, and 96 hours. Going down this, 10 *μ*L/well of CCK-8 proliferation reagent was added subsequently to all wells and further incubated for 2 hours at 37°C. Cellular optical densities were measured at 450 nm using a microplate reader (Thermo Fisher Scientific) [10].

### 2.7. Target Gene Analysis and Luciferase Gene Reporter Assay

The luciferase assay was conducted as narrated by Muramatsu et al. [11] and used to detect luciferase activity. HUVECs cells in the concentration of 5 × 10^4^ cells/well were loaded into the 96-well assay plate and cotransfected with pMIR construct (comprising WT or MUT 3′-UTR of SNHG1, miR-195-5p inhibitor or miR-195-5p mimics, pRL-TK *Renilla*, and VEGF-A) in the presence of lipofectamine 2000. Following 24 hours incubation, Luciferase Reporter System (Promega, USA) was used to assess pMIR-luciferase activity, following standard protocol.

### 2.8. Transwell Assay

The migratory ability of the cells was measured as described by Zhang et al. [12] using a Transwell Chamber (Millipore, MA, USA). Briefly, 5 × 10^4^ HUVECs containing DMEM (250 *μ*L) were loaded into the upper chamber following the addition of FBS (10%) into the lower chamber. Incubation was done at 37°C for 24 h with starved HUVECs following scraping of the cells adhered at the upper sides of the wells. HUVECs cells moved to the chamber below were adhered to methanol (20 minutes) and subsequently stained using 0.1% CV. Following staining for 20 minutes, imaging was performed with the help of a phase-contrast microscope (Olympus, Japan).

### 2.9. Capillary-Like Tube Formation Assay

Tube formation assay was executed following the method described by Zong et al. [13]. In short, a 96-well plate containing 50 *μ*L/well of Matrigel (Corning; Bedford, MA, USA) was incubated for 30 minutes at 37°C. Following incubation, HUVECs cells were loaded on the gel chambers and further maintained for 6 hours. For the analysis of tube length and number of branches, imaging was performed with the help of ImageJ software. The experiment was conducted in triplicate, and mean values with standard deviations were noted for each experiment.

### 2.10. Western Blot (WB) Analysis

Total protein isolation was performed in RIPA buffer following the detection of protein quality with a BCA protein assay kit (Beyotime, China). Following quantification, lysates were subjected to SDS-PAGE analysis and subsequently shifted to polyvinylidene fluoride (PVDF). Next, the following membrane was incubated at 4°C for 24 hours with the addition of primary antibodies and washed with tris-buffer saline. Following the manufacturer's instructions, membranes were shifted for second incubation for 1 hour with secondary antibodies. Results were visualized with a chemiluminescence western blotting kit (Global Life Sciences Solutions, USA).

### 2.11. Development of CRNV Rats

A properly established room with adequate temperature, light, ad libitum feeding, and humidity was used to house Wister rats. Intraperitoneal injection of pentobarbital containing 40 mg/kg dosage was used to anaesthetize rats. Alkali burn was induced by placing a small disc of filter paper soaked in 1 mM NaOH and subsequently washed with PBS (10 mL). Following alkali burn, rats were parted into two groups, named the control group (si-control) and experimental group (si-SNHG1). Slit-lamp microscopy results were used to measure the sizes of induced CRNV in rats at 3, 5, and 7 days following the sacrifice on day 7 for further experiments. The experiment in animals was approved by the Ethics Committee of People's Hospital of Shanxi Province.

### 2.12. Statistical Analysis

The experiments were conducted in set of three and presented as mean ± deviations from the standard value (SD). The significant difference was computed using SPSS 23.0 followed by Dunnett's test, ANOVA, and Pearson's correlation test.

## 3. Results

### 3.1. SNHG1 and VEGFA Were Overexpressed in the Tissues of CRNV and VEGF-Treated HUVECs

**Figure 1:**
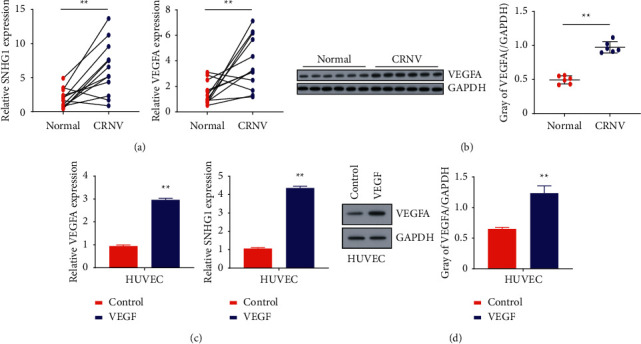
The expression pattern of SNHG1 and VEGF-A in corneal neovascularization tissues of the rat model: (a) the expression of SNHG1 was determined using real-time PCR, (b) the expression of VEGF-A was detected using real-time PCR and western blot analysis, (c) the expression of VEGF-A determined by qRT-PCR and western blot in HUVECs, and (d) relative expression of SNHG1 in HUVECs. ^*∗*^*P* < 0.01 versus control.

At first, the characteristics of the 12 vascularized corneas were observed: neovascularization with fibrous connective tissue hyperplasia in the periphery of the cornea, which is expressed as a mesh or curtain-like shape. Subsequently, the relative expression of SNHG1 and endothelial growth factor VEGF-A was detected in these tissues of CRNV and VEGF-treated HUVECs cells. Results from quantitative qRT-PCR and WB analysis revealed significantly upregulated levels of SNHG1 in CRNV tissues in contrast to the control group (Figures 1(a) and 1(b); *P* < 0.01). Next, the data from qRT-PCR and WB analysis demonstrated the expression level of mRNAs and protein of SNHG1 and VEGF-A was increased in VEGF-stimulated HUVECs cells (Figures 1(c) and 1(d); *P* < 0.01).

### 3.2. Knockdown of SNHG1 Lead to Suppression of Tube Formation, Migration, and Proliferative Abilities in HUVECs

**Figure 2:**
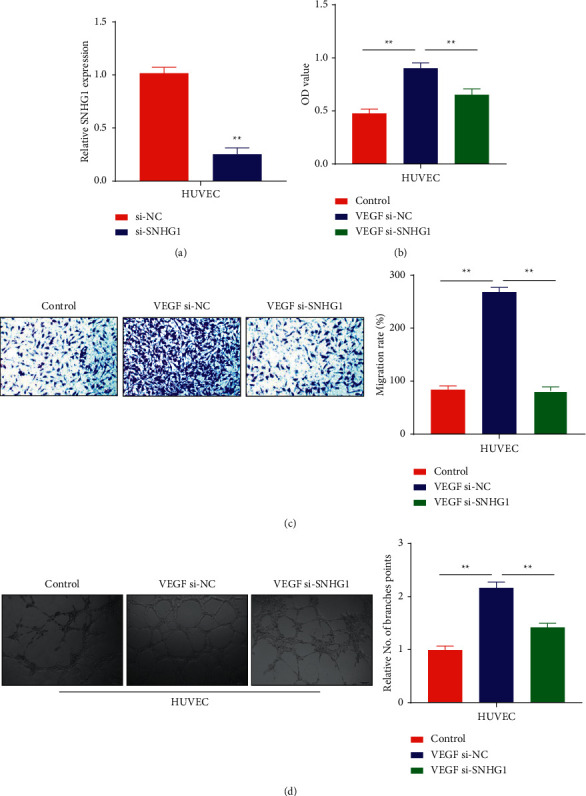
The expression patterns of SNHG1 and VEGF-A in HUVECs: (a) relative expression of SNHG1 in HUVECs transfected with si-SNHG1 measured through qRT-PCR, (b) OD values of VEGF-stimulated HUVECs cells measured through CCK-8 assay, (c) migration rate of HUVECs in VEGF-treated cells detected through transwell bioassay, and (d) tube forming ability of HUVECs in VEGF-treated cells detected through tube formation assay. ^*∗*^*P* < 0.01 versus control.

To explore the importance of SNHG1 in cell tube formation, proliferation, and migration of HUVECs, cells transfection was performed with SNHG1 following the establishment of knockdowns in HUVECs experimental group. The knockdown efficacy of SNHG1 in transfected cells was determined by qRT-PCR. When compared with si-RNA as the negative control group (si-NC), cells transfected with si-RNA of SNHG1 (si-SNHG1) showed considerable knockdown of lncRNA SNHG1 with statistically significant results (*P* < 0.01; Figure 2(a)). Results obtained were further confirmed by the CCK-8 method in which light absorption values at 450 nm were noted for all control and experimental groups. Consequences from the bioassay revealed that VEGF stimulation caused a remarkable promotion of cell proliferation, whereas si-SNHG1 transfection completely reversed the effects of VEGF (*P* < 0.01; Figure 2(b)). This suggests that VEGF stimulation could potentially increase cell migration and proliferation ability. Hence, the migratory ability of the cells was determined using transwell bioassay. Results from the assay indicated that VEGF stimulation notably increased the migratory ability of the cells transfected with si-NC and SNHG1 knockdown reversed the effects in HUVECs (*P* < 0.01; Figure 2(c)). Next, HUVECs cells were subjected to a tube formation assay to measure their angiogenic potential. The results were statistically compared in the control, si-NC, and si-SNHG1 groups after 6 hours of transfection. The data suggested that in comparison to the control group, the VEGF-stimulated si-NC group demonstrated notably high rates of tube formation; however, the VEGF-treated si-SNHG1 group showed decreased tube formation potential (*P* < 0.01; Figure 2(d)).

### 3.3. SNHG1 Is a Direct Regulator of MicroRNA-195-5p Expression

**Figure 3:**
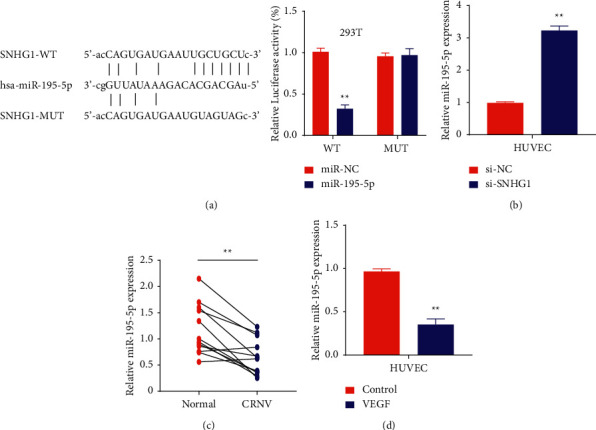
SNHG1 regulates the expression of miR-195-5p: (a) online prediction through StarBase for elucidation of SNHG1 binding site in miR-195-5p, relative luciferase activity of the cells cotransfected with miR-195-5p and wild-type and mutant SNHG1, (b) relative expression of miR-195-5p in HUVECs cotransfected with si-SNHG1, (c) relative expression of miR-195-5p in 12 pairs of patients with CRNV and normal individuals, and (d) relative expression of miR-195-5p in VEGF-stimulated HUVECs. ^*∗*^*P* < 0.01 versus control.

The results from the above experiments showed a positive correlation between VEGF and SNHG1expression that was further investigated by identifying the direct targets of SNHG1. To elucidate the targets of SNHG1 and understand the underlying mechanism, StarBase v2.0 was performed between SNHG1 and miRNAs that showed miR-195-5p as the potential target for SNHG1. The results were further elucidated by luciferase reporter gene assay in 293T cells. Results from the experiment clearly stated that 293T cells transfected with SNHG1wild-type (WT) + miR-NC increased luciferase activity, which disappeared in the cells with wild-type + miR-195-5p demonstrated that miR-195-5p can inhibit the luciferase activity in cells. However, the inhibition disappeared after mutation of predicted SNHG1 binding site (Figure 3(a)). Next, the qRT-PCR was carried out to investigate the miR-195-5p expression levels in HUVECs cells with SNHG1 knockdown. Results showed a very clear picture as expected, where SNHG1 knockdown increased the levels of miR-195-5p significantly (*P* < 0.01; Figure 3(b)). Also, dual verification of miR-195-5p expression was done in 12 pairs of healthy individuals and CRNV patients, which clearly showed the statistically reduced miR-195-5p expression in CRNV patients (*P* < 0.01; Figure 3(c)). Finally, the miR-195-5p levels were monitored by qRT-PCR in VEGF-stimulated HUVECs, which showed markedly decreased miR-195-5p values indicating the role of VEGF in the development of CRNV (*P* < 0.01; Figure 3(d)).

### 3.4. SNHG1-Regulated Corneal Angiogenesis by Reducing miR-195-5p

**Figure 4:**
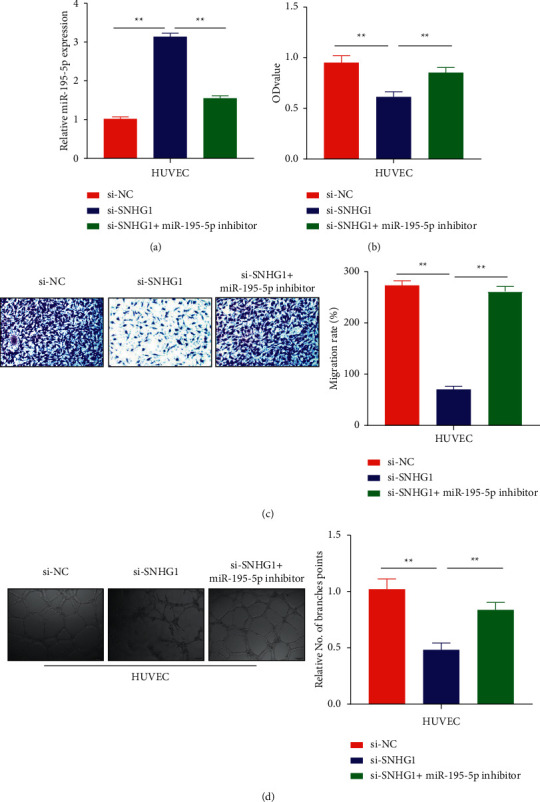
SNHG1 regulates corneal angiogenesis by sponging miR-195-5p: (a) relative miR-195-5p expression in HUVECs cotransfected with si-SNHG1 and si-SNHG1 + miR-195-5p inhibitor, (b) OD values of HUVECs cells measured through CCK-8 assay transfected with si-SNHG1 and si-SNHG1 + miR-195-5p inhibitor, (c) migratory ability of the cells transfected with si-SNHG1 and si-SNHG1 + miR-195-5p inhibitor detected through transwell assay, and (d) tube formation ability of the cells transfected with si-SNHG1 and si-SNHG1 + miR-195-5p inhibitor.

Findings of the data suggested the direct role of SNHG1 in regulating miR-195-5p levels and the development of corneal angiogenesis. Again, the data were validated to elucidate whether SNHG1 is vital in corneal angiogenesis; the miR-195-5p expression in three different groups including si-NC, si-SNHG1, and si-SNHG1 + miR-195-5p inhibitor was determined by qRT-PCR. Si-RNA SNHG1-transfected cells showed remarkably higher miR-195-5p expression levels as compared to the si-NC control group. However, cotransfecting si-SNHG1 + miR-195-5p inhibitor partially reverted the results in comparison to the si-SNHG1 group and demonstrated a partial decrease of the miR-195-5p in HUVECs cells (*P* < 0.01; Figure 4(a)). Proliferative efficiency of the cells was identified with the CCK-8 method of VEGF-stimulated HUVECs. Results from the experiment were in accordance with previous findings suggesting the role of SNHG1 in cellular angiogenesis. Light absorption values from the CCK-8 bioassay showed the highest values for the cells in the si-NC group. However, HUVECs transfected with si-SNHG1 displayed a clear decrease in light absorption suggesting that suppression of SNHG1 through si-SNHG1 can inhibit cell proliferation. Further validation was made by transfecting HUVECs with a si-SNHG1 + miR-195-5p inhibitor, which suggested little increase in light absorption values of the cells (*P* < 0.01; Figure 4(b)). Cellular migration was detected through transwell assay, where decreased migration was observed in the HUVECs transfected with si-SNHG1, while cells transfected with si-NC and si-SNHG1 + miR-195-5p inhibitor showed significant migration (*P* < 0.01; Figure 4(c)). Lastly, a few branches were observed in HUVECs transfected with the si-SNHG1 group, while more branches were observed in si-NC and si-SNHG1 + miR-195-5p inhibitor groups in tube bioassay, and the difference was statistically significant (*P* < 0.01; Figure 4(d)).

### 3.5. SNHG1 Increased the Level of VEGF-A through miR-195-5p

**Figure 5:**
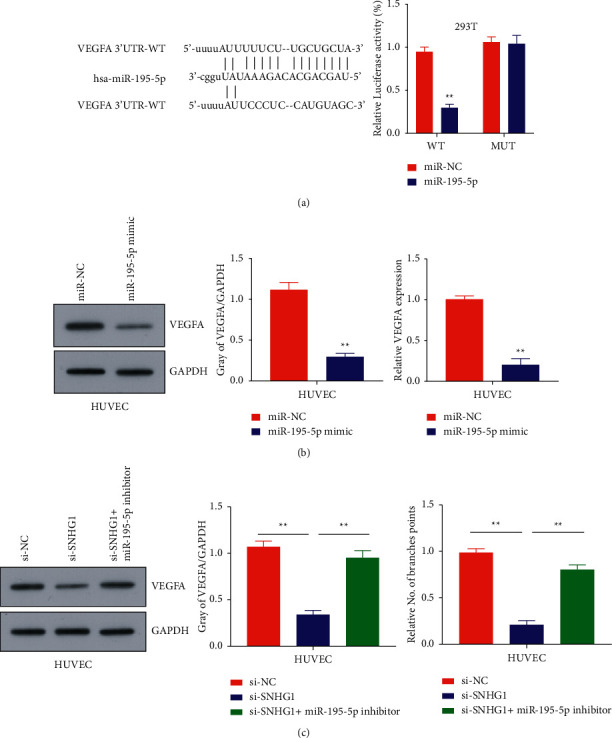
SNHG1 increased the level of VEGF-A through miR-195-5p: (a) online prediction through StarBase for elucidation of VEGF-A binding site in miR-195-5p, relative luciferase activity of the cells cotransfected with miR-195-5p and wild-type and mutant VEGF-A, (b) western blot and qRT-PCR analysis of HUVECs transfected with miR-195-5p mimic, relative VEGF-A expression in the HUVECs transfected with miR-195-5p mimic, and (c) western blot and qRT-PCR analysis of the HUVECs transfected with si-SNHG1 + miR-195-5p inhibitor.

To investigate the mechanism that how SNHG1 serves the role of a sponge for miR-195-5p, the binding sites of miR-195-5p in VEGF-A were determined by StarBase. The binding sites of miR-195-5p in VEGF-A were detected at the 3′UTR region of VEGF-A, which was validated after creating a mutant of VEGF-A. The results were further confirmed by determining relative luciferase activity of miR-NC and miR-195-5p groups in VEGF-A wild-type and VEGF-A mutant 293T cells. Results showed that overexpression of miR-195-5p can inhibit the luciferase activity in VEGF-A-treated cells, and the inhibitory effect disappeared after the predicted 3′-UTR binding site of VEGF-A was mutated (*P* < 0.01; Figure 5(a)). Next, WB analysis showed the expression levels of VEGF-A in HUVECs transfected with miR-NC and miR-195-5p mimic. Considerable high expression of VEGF-A was noted in the miR-NC group as compared to the cells transfected with miR-195-5p mimic indicating the potential of miR-195-5p mimic in controlling the expression of VEGF-A. GAPDH was run as a positive control for expression analysis (*P* < 0.01; Figure 5(b)). Finally, expression levels of VEGF-A mRNA and protein in different groups, si-NC, si-SNHG1, and si-SNHG1 + miR-195-5p inhibitor, in HUVECs cells were detected by qRT-PCR and WB analysis. In contrast to the si-NC group, both mRNA and protein of VEGFA were significantly decreased in the si-SNHG1 group, whereas si-SNHG1 mRNA and VEGF-A protein showed a considerably increased expression in HUVECs transfected with si-SNHG1 + miR-195-5p inhibitor, and the difference was statistically significant (*P* < 0.01; Figure 5(c)).

## 4. Discussion

Many studies in the past years showed that various factors can cause corneal injuries, including the inflammatory causes of neovascularization; for example, ocular mucous membrane pemphigoid (MMP) can induce an inflammatory response that leads to the formation of a fibrotic scar [14], Sjögren's syndrome can cause keratitis sicca with inflammation [15]. Moreover, ocular graft-versus-host disease affects all the structures of the entire ocular surface system, including lacrimal and meibomian glands, cornea, conjunctiva, eyelids, nasolacrimal duct, and tears [16]. However, the most common of factors that cause corneal injuries is the alkali burns, which finally lead to nonspecific keratitis [2]. Alkalis can easily penetrate corneal tissues absorbing water and completely dehydrate the cell leading to the death of corneal tissues. This in- turn stimulates corneal immune reactions, triggering damage to ocular and intraocular tissues [2, 3]. This situation often leads to the development of corneal ulcers including CRNV, finally causing blindness. CRNV indicates one of the severe eye problems in recent years and has been extensively studied for insights into disease progression [13]. Among popular methods and techniques to investigate the underlying CRNV disease mechanisms, alkali-burn corneal injuries have been extensively used. For instance, alkali-burn CRNV rat models were designed by Sun et al. [17] to study apoptosis and the effects of CRNV on cell cycle patterns. Moreover, inflammatory patterns, fibrosis, retinal damage, and oxidative stresses have been discussed in alkali-burn rats with CRNV [18–20]. This study demonstrates the in-vivo establishment of alkali-burn models in rats as an example to further investigate CRNV development and angiogenesis and the role of lncRNA SNHG1 in disease development. The study demonstrated the involvement and significance of VEGF in the progress of CRNV. VEGF has been shown as a direct stimulator in angiogenesis and identified as a therapeutic target by developing certain anti-VEGF growth factors [21]. In this study, the HUVEC-based CRNC model was constructed to investigate the effects of VEGF stimulation on CRNV angiogenesis.

Accumulating shreds of evidence has reported connections between lncRNAs in CRNV malignancy and angiogenesis. For instance, upregulated levels of lncRNA NEAT1 were observed in the progression of CRNV and promotion of inflammatory responses [22]. Similarly, lncRNA MIAT suppression alleviated CRNV through the regulation of miR-1246/ACE [23]. Likewise, IL-6 relevant lncRNA profiles have been investigated in inflammatory and tumor diseases including CRNV [24]. Likewise, lncRNA H19 was demonstrated for its role in the negative regulation of corneal epithelial adhesion and cell proliferation [25]. Similarly, SNHG1 has been shown as a contributor to gastric cancer proliferation through DNMT1 [26]. Upregulated SNHG1 was demonstrated as a key agent in lung cancer development via the inhibition of miR-101-3p [27]. Moreover, it was also seen that SNHG1 enhances invasiveness and proliferation in cervical cancer [28] However, the potential of SNHG1 is still elusive. Nonetheless, its detailed role in CRNV angiogenesis has been exclusively studied in this study. Not only the upregulated levels of SNHG1 were observed in VEGF-stimulated HUVECs, but also its knockdown notably reduced migration, proliferation, and tube formation in HUVECs. Increased branch points and blood vessels were observed in the rats with CRNV as compared to control groups without CRNV. This research unveils the effects and expression of lncRNA SNHG1 on the CRNV angiogenesis as ceRNA to sponge miR-195-5p.

In the first set of experiments, expression levels of SNHG1 and VEGF-A were observed in developed models and VEGF-stimulated HUVECs through qRT-PCR and WB analysis. The results manifested overexpressed SNHG1 and VEGF-A in CRNV samples from HUVECs, suggesting the argument that SNHG1 can be aberrantly expressed in CRNV and thus can promote angiogenesis.

Bioinformatics studies to determine the potential target of SNHG1 showed a direct association with miR-195-5p, and SNHG1 acts as a sponge for this microRNA (StarBase database; https://www.starbase.sysu.edu.cn/). StarBase showed a clear binding site for miR-195-5p, which was further validated by creating a mutant of SNHG1. This is a well-documented mechanism where lncRNAs bind microRNAs to inhibit their regulatory activities and contribute to disease development [22]. In this case, luciferase reporter activity inhibited in the cells transfected with miR-195-5p, whereas vice-versa results were obtained after SNHG1-MUT was used to transfect the cell line. Moreover, the CCK-8 assay to analyze cell proliferation confirmed the highest expression of miR-195-5p in the cells transfected with si-SNHG1 as compared to si-SNHG1 + miR-195-5p inhibitor and si-NC groups. Later on, data from transwell assay and tube formation bioassay confirmed the results confirming miR-195-5p as the direct target of SNHG1. The results are in accordance with previously reported literature, where inhibition or downregulation of lncRNA significantly reduces the proliferative and metastatic abilities of tumor cells leading to the suppression of angiogenesis [20–26].

## 5. Conclusions

Overall, the study demonstrates the aberrant expression of SNHG1 for the first time in patients suffering from CRNV and its significance in central angiogenesis. The study showed that SNHG1 inhibition significantly reduced cell proliferation, migration, and tube formation in HUVECs transfected with lncRNA SNHG1. Mechanistic insights into the SNHG1 showed that SNHG1 acts as a sponge for miR-195-5p and upregulates the levels of VEGF-A. These findings demonstrate SNHG1 as a likely therapeutic target for the treatment of CRNV.

## Data Availability

The data underlying this article are available from the corresponding author upon request.
